# Taste responsiveness of Western chimpanzees (*Pan troglodytes verus*) to five food-associated saccharides

**DOI:** 10.1007/s10329-018-0697-0

**Published:** 2018-11-15

**Authors:** Ellen Norlén, Desirée Sjöström, Madeleine Hjelm, Therese Hård, Matthias Laska

**Affiliations:** 1 IFM Biology, Linköping University, 581 83 Linköping, Sweden; 2 Borås Zoo, 501 13 Borås, Sweden

**Keywords:** Taste preference thresholds, Relative sweetness, Taste difference thresholds, Food-associated saccharides, Western chimpanzees, *Pan troglodytes verus*

## Abstract

Using a two-bottle choice test of short duration, we determined taste preference thresholds for sucrose, fructose, glucose, lactose, and maltose in three Western chimpanzees (*Pan troglodytes verus*). Further, we assessed relative preferences for these five saccharides when presented at equimolar concentrations and determined taste preference difference thresholds for sucrose, that is, the smallest concentration difference at which the chimpanzees display a preference for one of the two options. We found that the chimpanzees significantly preferred concentrations as low as 20 mM sucrose, 40 mM fructose, and 80 mM glucose, lactose, and maltose over tap water. When given a choice between all binary combinations of these five saccharides presented at equimolar concentrations of 100, 200, and 400 mM, respectively, the animals displayed significant preferences for individual saccharides in the following order: sucrose > fructose > glucose = maltose = lactose. The taste difference threshold for sucrose, expressed as Weber ratio (ΔI/I), was 0.3 and 0.4, respectively, at reference concentrations of 100 and 200 mM. The taste sensitivity of the chimpanzees to the five saccharides falls into the same range found in other primate species. Remarkably, their taste preference thresholds are similar, and with two saccharides even identical, to human taste detection thresholds. The pattern of relative taste preferences displayed by the chimpanzees was similar to that found in platyrrhine primates and to the pattern of relative sweetness as reported by humans. Taken together, the results of the present study are in line with the notion that taste sensitivity for food-associated carbohydrates may correlate positively with phylogenetic relatedness. Further, they support the notion that relative preferences for food-associated carbohydrates, but not taste difference thresholds, may correlate with dietary specialization in primates.

## Introduction

Comparative studies of taste perception allow us to gain insight into the mechanisms underlying the evolution of the sense of taste and the causes of between-species differences in taste performance. Primates are a particularly suitable taxon in this respect, as they comprise a large variety of dietary specializations (Fleagle 2013) and the composition of food is commonly thought to affect the taste perception of a given species (Dominy et al. 2001). Accordingly, the sense of taste has been studied with regard to its anatomy (e.g., Hofer et al. 1990; Emura 2017; Pastor et al. 2017), electrophysiology (e.g., Hellekant et al. 1996, 1997, 1998), and genetics (e.g., Go et al. 2005; Wooding et al. 2006; Li et al. 2011) in all major taxa of primates, including the hominids. Surprisingly little, in contrast, is known about taste perception in the Great Apes at the behavioral level. With regard to substances tasting sweet to humans, for example, taste preference thresholds in the chimpanzee have so far only been reported for fructose (Simmen and Charlot 2003; Remis 2006), and for the two sweet-tasting compounds of *Stevia rebaudiana*, stevioside and rebaudioside A (Nicklasson et al. 2018).

Chimpanzees (*Pan troglodytes*) include a considerable proportion of fruit into their diet, with values ranging from 55 to 75% of their annual intake, depending on study site, season of the year, and recording method (e.g., Head et al. 2011; McLennan 2013; Morgan and Sanz 2006; Watts et al. 2012; Wrangham et al. 1998). Accordingly, they are often referred to as primarily frugivorous and as ripe fruit specialists (Milton 1999; Pruetz 2006). Their food selection behavior suggests that chimpanzees may use the sweetness of fruits as a criterion for consumption (Dominy et al. 2016; Hladik and Simmen 1996).

Fruits contain a variety of soluble carbohydrates that taste sweet to humans (Kinghorn and Soejarto 1986). However, the disaccharide sucrose and its monosaccharide components fructose and glucose are usually quantitatively predominant in fruits and account for more than 90% of their total carbohydrate content (Nagy and Shaw 1980). The disaccharide lactose is the main carbohydrate in milk, the first diet in the life of mammals (Jensen 1995), and the milk of chimpanzees, for example, contains 7.4 g/100 ml of lactose (Hinde and Milligan 2011). The disaccharide maltose originates in considerable amounts from the enzymatic degradation of starch during mastication, and thus contributes to the taste sensation while feeding on starch-containing plants (Butterworth et al. 2011).

It was therefore the aim of the present study to assess the taste responsiveness of Western chimpanzees to these five food-associated carbohydrates. More specifically, we (1) determined taste preference thresholds for sucrose, fructose, glucose, lactose, and maltose in *P. troglodytes verus*, (2) assessed relative preferences of the chimpanzees for these five saccharides when presented at equimolar concentrations, and (3) determined taste preference difference thresholds for sucrose, that is, the smallest concentration difference for which the chimpanzees displayed a reliable difference in preference. To this end, we employed a two-bottle preference test of short duration (Richter and Campbell 1940). This test allows us to measure both absolute and relative preferences for taste substances and, at the same time, largely rules out the influence of postingestive factors on an individual’s ingestive behavior.

## Methods

### Animals

We assessed taste responsiveness in two adult female and one adult male Western chimpanzees (*P. troglodytes verus*). The animals were 27, 33, and 48 years old at the start of the study. They were housed, together with one other individual, at Borås Zoo, Sweden, in a 750 m^3^ indoor exhibit, with access to a 560 m^2^ outdoor island with natural vegetation. We performed the tests in a smaller room adjacent to the indoor exhibit, which held three compartments in which the animals were tested separately to avoid competition and distraction. All three animals were trained to voluntarily enter the test compartments and were completely accustomed to the procedure described below (Nicklasson et al. 2018). The animals were fed fresh fruit and vegetables (e.g., apples, bananas, grapes, melons, mangoes, figs, tomatoes, cucumber, carrots, broccoli, avocado, with seasonal variations) three times per day. Commercial primate chow pellets were served once per day, eggs were served weekly, and water was provided ad libitum. The amount of food offered daily to the animals was such that leftovers were still present on the floor the next morning. Thus, it was unlikely that ravenous appetite affected the animals’ ingestive behavior.

### Ethical note

The experiments reported here comply with the *American Society of Primatologists’ Principles for the Ethical Treatment of Primates,* and also with current Swedish laws. This study was approved by Gothenburg’s Animal Care and Use Committee (Göteborgs djurförsöksetiska nämnd, protocol #75-2016).

### Taste stimuli

We used the following five saccharides: sucrose (CAS# 57-50-1), fructose (CAS# 57-48-7), glucose (CAS# 50-99-7), maltose (CAS# 6363-53-7), and lactose (CAS# 63-42-3). All substances were obtained from Sigma-Aldrich (St. Louis, MO, USA) and were of the highest available purity (≥ 99.5%).

### Procedure

We used a two-bottle preference test of short duration (Richter and Campbell 1940). The animals were allowed to drink for 1 min from a pair of simultaneously presented graduated cylinders of 700 ml with metal drinking spouts. We performed between four and six of such 1-min trials per day and animal, with usually two trials in the morning, around noon, and in the afternoon, respectively.

### Determination of taste preference thresholds

To determine taste preference thresholds, the animals were given the choice between tap water and defined concentrations of a saccharide dissolved in tap water. With all five saccharides, testing started at a concentration of 200 mM and proceeded in the following steps (100, 50, 20, 10 mM, etc.) until an animal failed to show a significant preference. Subsequently, they were presented with intermediate concentrations (between the lowest concentration that was preferred and the first concentration that was not) in order to determine the preference threshold value more exactly. The order in which the five saccharides were tested was the same for all three animals: (1) sucrose, (2) fructose, (3) glucose, (4) maltose, and (5) lactose.

### Assessment of relative taste preferences

To assess relative preferences, the animals were given the choice between two saccharide solutions presented at equimolar concentrations. All ten possible binary stimulus combinations (e.g., sucrose vs. fructose, maltose vs. lactose, etc.) were tested. To assess whether relative preferences were stable at different concentrations, three series of tests were performed at 100, 200, and 400 mM, respectively.

### Determination of taste preference difference thresholds

To determine taste preference difference thresholds for sucrose, the animals were given the choice between a reference concentration and lower concentrations of the same substance until the animals failed to show a significant preference for one of the two alternatives. To assess whether taste preference difference thresholds were stable at different concentrations, two series of tests were performed, using reference concentrations of 100 mM and 200 mM sucrose, respectively. To this end, the 100 mM sucrose reference concentration was tested against sucrose solutions of 20, 50, 60, 70, 80, and 90 mM, respectively, and the 200 mM sucrose reference concentration was tested against sucrose solutions of 20, 50, 90, 100, 120, 130, 140, and 150 mM, respectively.

In all three experiments, we presented each pair of stimuli ten times per individual animal, and the position of the stimuli was pseudo-randomized in order to counterbalance possible position preferences. Care was taken that an animal sampled both stimuli at least once during each trial. To maintain the animals’ motivation and willingness to cooperate, testing of the different stimulus combinations within an experiment did not follow a strict order but was pseudo-randomized. This was true both within a given session (morning, noon, or afternoon) and between sessions.

### Data analysis

For each animal, we recorded the amount of liquid consumed from each bottle, summed it for the ten trials with a given stimulus combination, converted it to percentages (relative to the total amount of liquid consumed from both bottles), and took 66.7% (i.e., 2/3 of the total amount of liquid consumed) as the criterion of preference. We chose this rather conservative criterion for reasons of comparability of data as the same criterion had been used in previous studies on sweet-taste responsiveness with other primate species (Laska 1996, 1997, 2000; Laska et al. 1996, 1998, 1999a, b, 2001; Nicklasson et al. 2018; Wielbass et al. 2015), and in order to avoid misinterpretation due to a too liberal criterion. Additionally, we performed binomial tests, and regarded an animal as significantly preferring one of the two stimuli if it reached the criterion of 66.7% and consumed more from the bottle containing the preferred stimulus in at least eight out of ten trials (binomial test, *P* < 0.05).

Thus, we defined taste preference threshold as the lowest concentration at which the animals met both criteria mentioned above. Preliminary analyses of the data indicated that there were no systematic differences in choice behavior and liquid consumption between the first and the second presentation of a session, or between the morning, the noon, and the afternoon session, respectively. Intraindividual variability of the amount of liquid consumed across the ten trials with a given stimulus combination was low and averaged less than 20%. Thus, a theoretically possible bias in the overall preference score due to excessive drinking in aberrant trials did not occur. All data are reported as mean values ± SD.

Taste preference difference threshold values were expressed as Weber ratios (ΔI/I). They are based on the Weber–Fechner law of psychophysics and commonly used to quantify the just noticeable difference (JND) between different concentrations of the same stimulus (Fischer et al. 1965).

## Results

### Taste preference thresholds

**Fig. 1:**
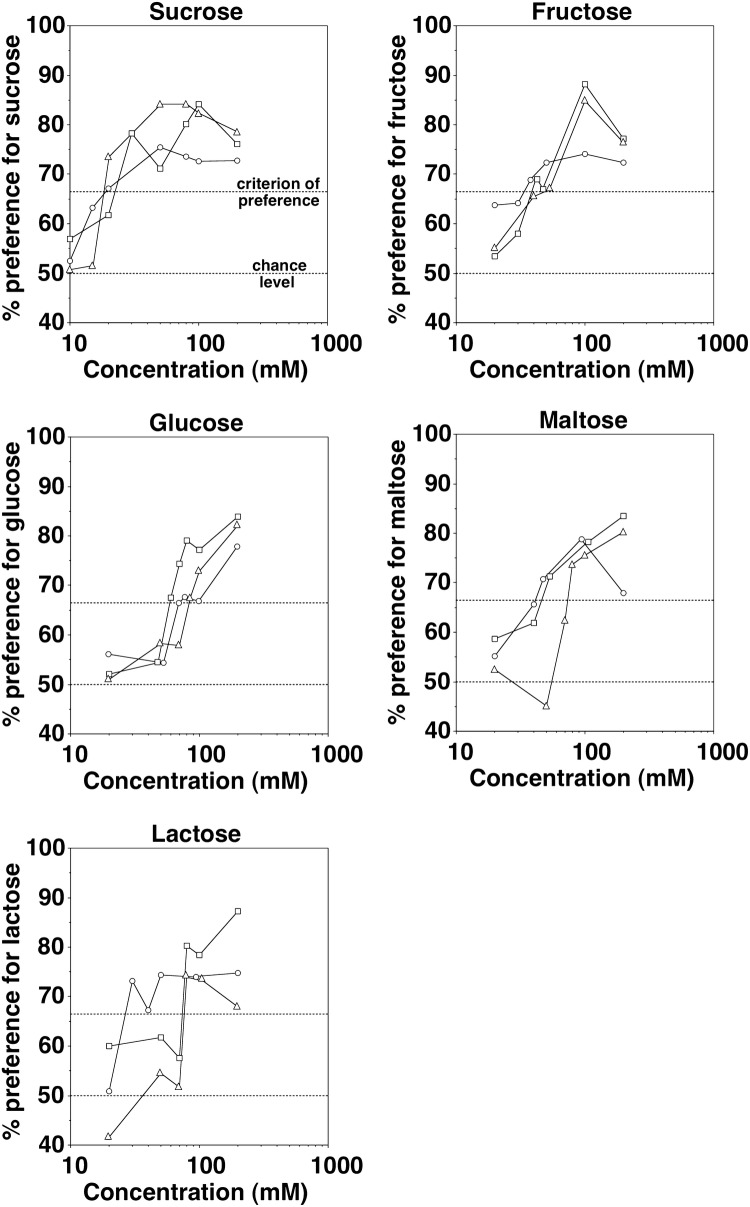
Mean taste responses (± SD) of three Western chimpanzees to aqueous solutions of sucrose, fructose, glucose, maltose, and lactose tested against tap water. Each data point represents the mean value of ten trials of 1 min per animal. The dotted horizontal lines at 66.7% and at 50% indicate the criterion of preference and the chance level, respectively

Taste preference thresholds of the three Western chimpanzees were found to be 20 mM for sucrose, 40 mM for fructose, and 80 mM for glucose, maltose and lactose, respectively (Fig. 1). All animals failed to show a significant preference for the lowest concentrations presented, suggesting that the preference for higher concentrations was indeed based on the chemical nature of the stimuli. In most cases, interindividual variability was low for both sub- and suprathreshold concentrations tested and with only two exceptions all three animals either reached the criterion of preference (> 66.7% of total consumption, plus binomial test, *P* < 0.05) with a given stimulus combination or all three animals failed to do so.

### Relative taste preferences

**Fig. 2:**
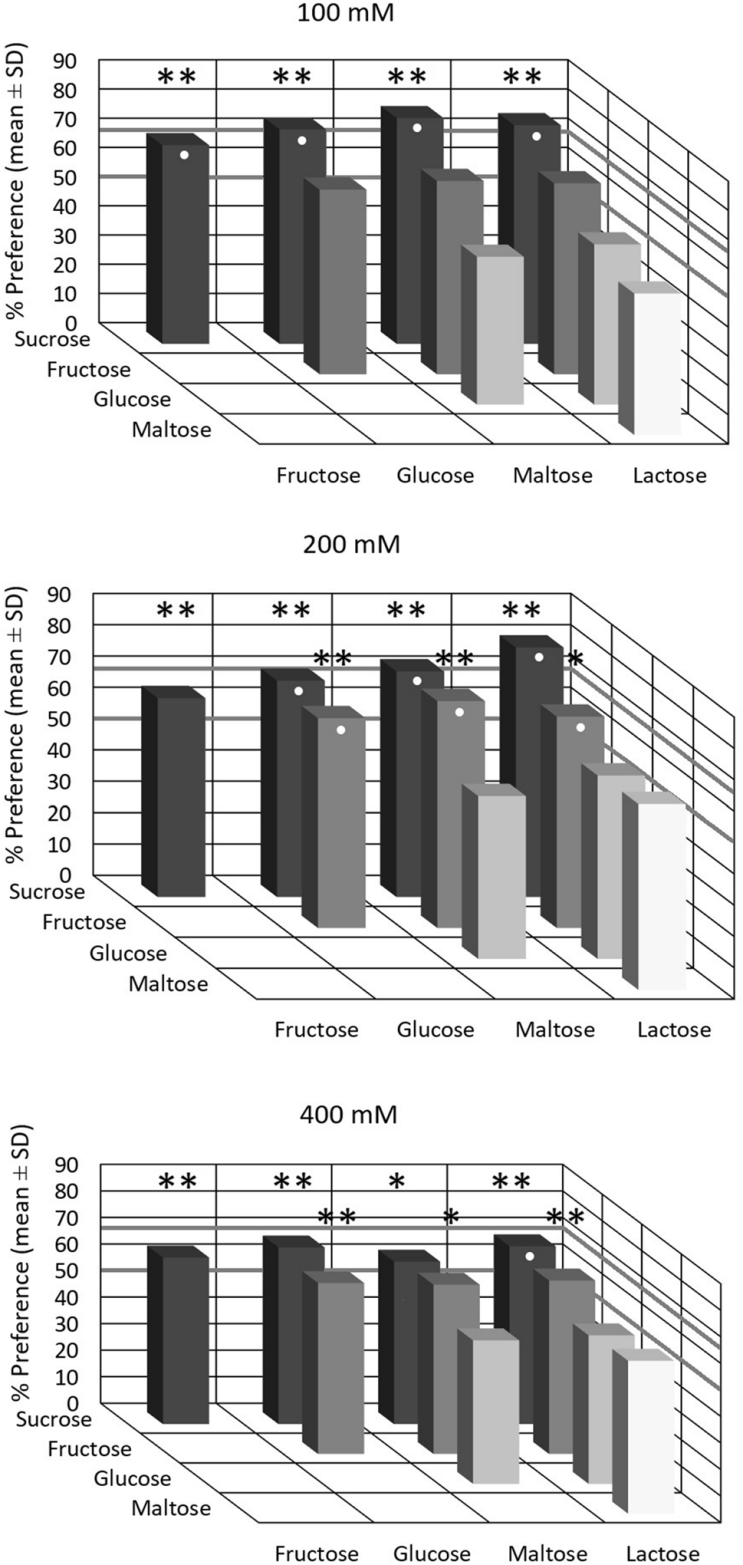
Relative taste preferences of three Western chimpanzees when given the choice between two aqueous solutions presented at equimolar concentrations of 100, 200, and 400 mM, respectively. Each bar represents the mean preference (± SD) from ten trials of 1 min per individual for the saccharide on the left side relative to the saccharide on the right. A white circle indicates a significant preference according to the criterion > 66.7% of total amount of liquid consumed. Asterisks indicate a significant preference according to a two-tailed binomial test, with *P* < 0.05 (one asterisk) and *P* < 0.01 (two asterisks). The fat horizontal lines at 66.7% and at 50% indicate the criterion of preference and the chance level, respectively

When given the choice between two aqueous saccharide solutions presented at equimolar concentrations of 100 mM, the chimpanzees significantly preferred sucrose over all other saccharides. Further, they showed a non-significant trend for preferring fructose over the other three saccharides (Fig. 2, upper panel). At 200 mM, the chimpanzees significantly preferred sucrose and fructose over glucose, lactose, and maltose (Fig. 2, middle panel). At 400 mM, the chimpanzees only displayed a significant preference for sucrose over lactose. However, they also showed a non-significant trend for preferring sucrose over glucose and maltose, and for preferring fructose over glucose, maltose, and lactose (Fig. 2, lower panel). Interindividual variability was low and with only few exceptions all three animals either reached the criterion of preference (> 66.7% of total consumption, plus binomial test, *P* < 0.05) with a given stimulus combination or all three animals failed to do so.

### Taste preference difference thresholds

**Fig. 3:**
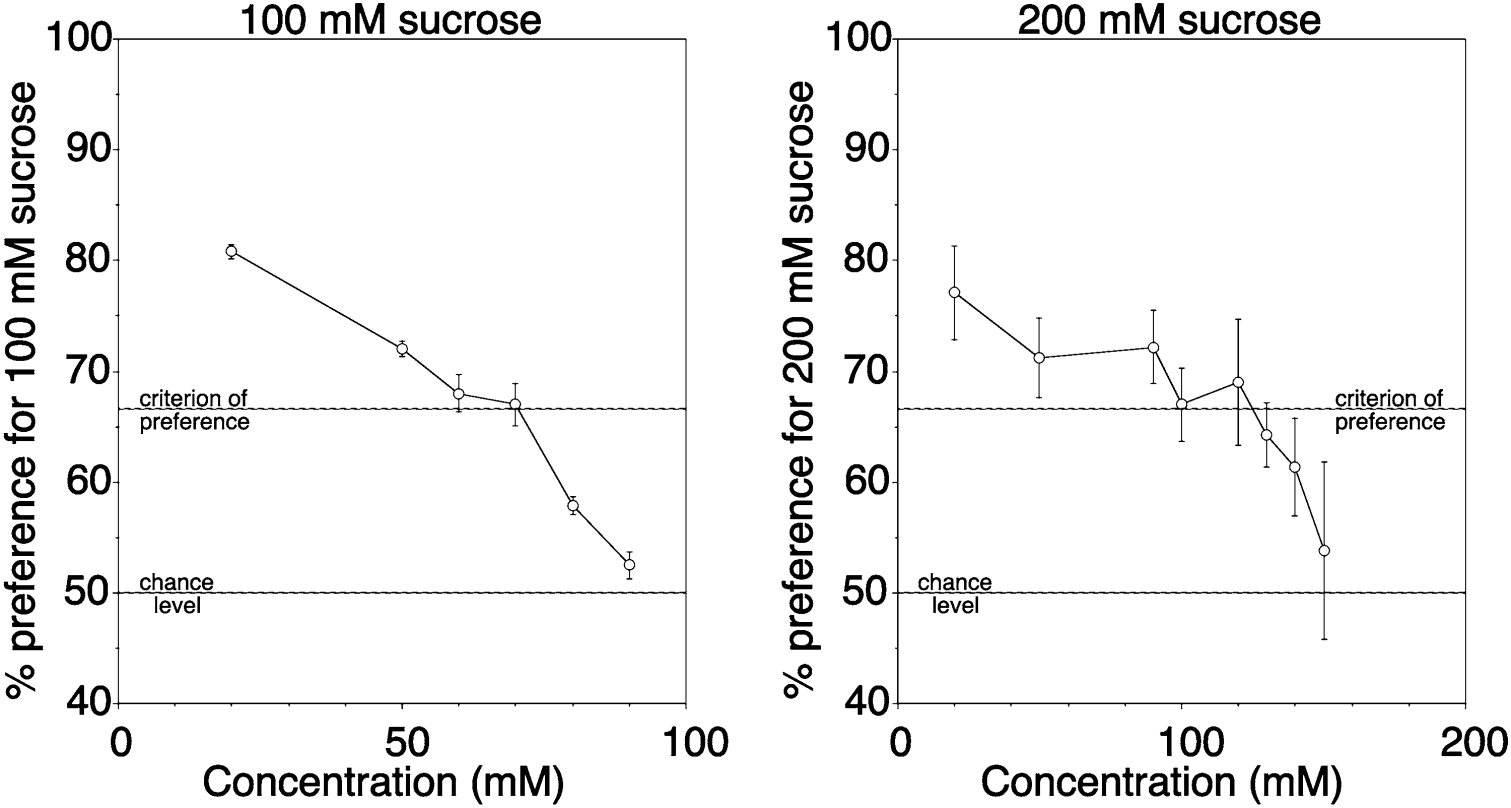
Taste preference difference thresholds at two different reference concentrations of sucrose (100 and 200 mM) in three Western chimpanzees. Each data point represents the mean value (± SD) of ten trials of 1 min per animal. The dotted horizontal lines at 66.7% and at 50% indicate the criterion of preference and the chance level, respectively. Please note that, for reasons of readability of the graph, the *x*-axis displays concentrations on a linear rather than on a logarithmic scale

When given the choice between different concentrations of sucrose, the chimpanzees significantly preferred the reference concentration of 100 mM over concentrations up to 70 mM, and they significantly preferred the reference concentration of 200 mM over concentrations up to 120 mM (Fig. 3). Accordingly, the taste preference difference threshold for sucrose, expressed as Weber ratio (ΔI/I), was 0.3 at a reference concentration of 100 mM, and 0.4 at a reference concentration of 200 mM. Here, too, interindividual variability was low and with only few exceptions all three animals either reached the criterion of preference (> 66.7% of total consumption, plus binomial test, *P* < 0.05) with a given stimulus combination or all three animals failed to do so.

## Discussion

The results of the present study show that Western chimpanzees have a well-developed sensitivity for food-associated saccharides. Further, they show that the five carbohydrates differ in their attractiveness and thus in their relative sweetness and that *P. troglodytes verus* has a well-developed ability to distinguish between different concentrations of sucrose.

### Taste preference thresholds

The concentrations of sucrose, fructose, and glucose found in ripe tropical fruits are markedly higher than the taste preference thresholds of the chimpanzees found in the present study (Nagy and Shaw 1980). Thus, these saccharides should contribute substantially to the taste sensation in chimpanzees while feeding on fruits. This, in turn, supports the notion that chimpanzees may indeed use the sweetness of fruits as a criterion for consumption (Dominy et al. 2016; Hladik and Simmen 1996). Similarly, the lactose content of chimpanzee milk of 7.4 g/100 ml (Hinde and Milligan 2011), corresponding to a concentration of 217 mM, is markedly higher compared to their taste preference threshold of 80 mM found here. This suggests that infant chimpanzees experience sweet taste from their very first meal of breast milk on. Some authors even hypothesize this early exposure to the sweet taste of lactose in breast milk to be the reason for the “sweet tooth”, that is, the predilection for sweet taste in primates (Ramirez 1990; Beauchamp and Mennella 2009). Although it is not possible to reliably determine the concentration of maltose that is produced via the enzymatic degradation of starch during mastication, chimpanzees are known to produce salivary α-amylase (Perry et al. 2007) and are thus, unlike some other primate species, able to enzymatically convert starch to maltose. This, in turn, makes it likely that maltose also contributes to sweet-taste perception in chimpanzees while feeding on starch-containing fruits.

The taste preference threshold value of 40 mM for fructose determined in the present study compares favorably with the results of the only two previous studies which assessed sweet-taste sensitivity in chimpanzees with carbohydrates so far: Simmen and Charlot (2003) reported a taste preference threshold value of 40 mM for fructose in two chimpanzees, and Remis (2006) a value of 50 mM in four chimpanzees. This suggests that our results are robust, despite the limited number of animals used.

The taste sensitivity of *P. troglodytes verus* for the five saccharides tested here falls into the range of threshold values reported in other species of nonhuman primates that have been tested previously with the same or a similar method (two-bottle preference test of short duration) (Table 1). The pattern of sweet-taste sensitivity found in the chimpanzees, with the threshold value for sucrose being lowest among the five saccharides tested, and the threshold value for fructose being lower than that for glucose, is also consistent with the majority of nonhuman primates tested so far.

**Table 1:** Taste preference thresholds (in mM) for food-associated saccharides in primates

Species	Sucrose	Fructose	Glucose	Maltose	Lactose	References
Hominid primates						
* Pan troglodytes verus*	20	40	80	80	80	[1]
*Homo sapiens*	10	40	80	31	72	[2]
*Gorilla gorilla*		75				[3]
*Pongo pygmaeus*		15				[3]
Catarrhine primates						
*Macaca nemestrina*	10	20	20	10	30	[4]
*Macaca mulatta*	6					[5]
*Macaca radiata*	10			10		[6]
*Macaca fuscata*	10			10		[7]
*Papio hamadryas anubis*	10	20	25	20	20	[8]
*Cercopithecus pygerythrus*	11					[5]
*Cercopithecus nictitans*	11					[5]
Platyrrhine primates						
*Ateles geoffroyi*	3	15	20	20	10	[9]
*Saimiri sciureus*	10	40	90	90	100	[10]
*Saguinus midas niger*	66	66	330		250	[5]
*Saguinus fuscicollis*	50					[5]
*Saguinus oedipus*	125	16				[5,11]
*Cebuella pygmaea*	33	50	100		125	[5]
*Callithrix jacchus*	25	29.5				[5,12]
*Callithrix geoffroyi*		41				[11]
*Callithrix argentata*		19.5				[11]
*Leontopithecus rosalia*		19.5				[11]
*Leontopithecus chrysomelas*		21.5				[11]
*Callimico goeldii*		31				[11]
*Cebus apella*	8					[11]
*Aotus trivirgatus*	17					[5]
Strepsirrhine primates						
*Varecia variegata variegata*	25	25	50	50	50	[13]
*Eulemur coronatus*		21				[11]
*Eulemur fulvus*	9	22.5				[11]
*Eulemur macaco*	8	14				[11]
*Eulemur mongoz*	125	110				[5]
*Hapalemur simus*	17.5	18.5				[11]
*Hapalemur griseus*		16.5				[11]
*Phaner furcifer*	65					[11]
*Microcebus murinus*	167	47.5				[5, 14]
*Microcebus coquereli*	90					[11]
*Cheirogaleus major*	50					[11]
*Cheirogaleus medius*	143					[5]
*Propithecus verreauxi*	52.5					[11]
*Loris tardigradus*	50					[5]
*Nycticebus coucang*	330					[5]
*Galago senegalensis*	66					[5]

[1] present study; [2] van Gemert (2011); [3] Simmen and Charlot (2003); [4] Laska (2000); [5] Glaser (1986); [6] Sunderland and Sclafani (1988); [7] Nishi et al. (2016); [8] Laska et al. (1999a, b); [9] Laska et al. (1996); [10] Laska (1996); [11] Simmen and Hladik (1998); [12] Simmen (1994); [13] Wielbass et al. (2015); [14] Simmen et al. (1999)

It is interesting to note that the taste preference thresholds reported here with chimpanzees are also similar, and in two cases (fructose and glucose) even identical to the taste detection threshold values obtained with human subjects (Table 1). This is not trivial, as the sophisticated signal detection methods employed in taste tests with human subjects are known to yield lower threshold values compared to the simple two-bottle preference test used with nonhuman primates, which provides only a conservative approximation of a species’ taste sensitivity (Spector 2003). The finding of similar sweet-taste thresholds in chimpanzees and humans as well as the similarity in the pattern of sweet-taste sensitivity in *P. troglodytes verus* and *Homo sapiens* across the five saccharides supports the notion that phylogenetic relatedness may correlate positively with taste sensitivity for food-associated carbohydrates (Glaser et al. 1995; Nofre et al. 1996).

### Relative taste preferences

The food-associated saccharides used here are known to differ in their stimulating efficiency, that is, some of them (e.g., sucrose) are perceived by humans as sweeter than others (e.g., lactose) when presented at equimolar concentrations (Pfaffmann et al. 1971). Differences in ligand affinity to the mammalian sweet-taste receptor have been identified as the underlying molecular mechanism of this phenomenon (Chandrashekar et al. 2006). It is commonly agreed that differences in the attractiveness of sweet-tasting substances reflect differences in stimulating efficiency. This notion is supported by electrophysiological findings in nonhuman primates including chimpanzees (Scott et al. 1991; Plata-Salaman et al. 1993; Hellekant et al. 1997). Therefore, the relative taste preferences found here can be considered as an approximation of the relative sweetness as perceived by the chimpanzees. The pattern of relative taste preference found in the present study in Western chimpanzees (sucrose > fructose > glucose = maltose = lactose) is identical or at least largely similar to that found in previous studies with squirrel monkeys, spider monkeys, and black-and-white ruffed lemurs, and also similar to the pattern of relative sweetness as reported by human subjects (Table 2). All five species clearly prefer sucrose over the other saccharides tested when presented at equimolar concentrations. Interestingly, pigtail macaques differ markedly in their relative taste preferences for food-associated saccharides. Unlike the other primates tested so far, but similar to rats, *Macaca nemestrina* clearly prefers maltose over sucrose. It has been hypothesized that rodents such as rats, and possibly also macaques, may have an additional taste receptor for starch-derived polysaccharides that also responds to maltose, the disaccharide building-block of starch, but not to sucrose (Bachmanov and Beauchamp 2007). The diet of rats and pigtail macaques is known to contain a high proportion of starch, either in the form of seeds or in the form of starch-containing underground storage organs such as roots, tubers, and bulbs (Caldecott 1986). The diet of other primates such as chimpanzees, in contrast, usually does not contain significant quantities of starch. The present results are in line with this hypothesis. Thus, the present findings lend further support to the notion that relative preferences for food-associated carbohydrates may correlate with dietary specialization in primates.

**Table 2:** Relative taste preferences for food-associated saccharides in primates and the rat

Species	Relative taste preference	References
Hominid primates		
*Pan troglodytes verus*	Sucrose > fructose > glucose = maltose = lactose	[1]
*Homo sapiens*	Sucrose > fructose > maltose ≥ glucose ≥ lactose	[2]
Catarrhine primates		
*Macaca nemestrina*	Maltose > sucrose > glucose ≥ fructose ≥ lactose	[3]
Platyrrhine primates		
*Saimiri sciureus*	Sucrose > fructose > glucose ≥ maltose ≥ lactose	[4]
*Ateles geoffroyi*	Sucrose > fructose > glucose ≥ lactose ≥ maltose	[5]
Strepsirrhine primates		
*Varecia v. variegata*	Sucrose > fructose > glucose ≥ maltose ≥ lactose	[6]
Non-primate mammals		
*Rattus norvegicus*	Maltose > sucrose = glucose > lactose	[7]
*Rattus norvegicus*	Maltose > sucrose > glucose = fructose	[8]

[1] present study; [2] Pfaffmann et al. (1971); [3] Laska (2000); [4] Laska (1997); [5] Laska et al. (1998); [6] Wielbass et al. (2015); [7] Richter and Campbell (1940); [8] Sclafani and Mann (1987)

### Taste preference difference thresholds

For species which at least partly meet their energy requirements by using easily metabolizable carbohydrates, it should be particularly important to be able to evaluate the sugar content of potential food items. In addition to a sufficiently high sensitivity allowing for the detection of sugars in food, such species should have a well-developed ability to distinguish between different sugar concentrations in order to optimize their energy yield as the carbohydrate content and thus the nutritive value of fruits may change markedly during maturation (Kinghorn and Soejarto 1986). The food selection behavior of chimpanzees supports this notion, as they have been observed to regularly base their decisions about consumption or rejection of a fruit on the taste sensation they experience during the first bite (Hladik and Simmen 1996). The taste preference difference thresholds of the chimpanzees for sucrose found in the present study are in the same range as those reported in squirrel monkeys, but higher than those in spider monkeys and olive baboons (Table 3). This finding is somewhat unexpected, as some studies suggest that the ability to distinguish between different concentrations of sweet-tasting food constituents should correlate positively with the degree of frugivory (Laska et al. 1999a, b). Spider monkeys, for example, are clearly more frugivorous than squirrel monkeys and *Ateles geoffroyi* also displays lower Weber ratios, that is, a higher ability to discriminate between sucrose concentrations than *Saimiri sciureus*. However, such a correlation might only hold true for platyrrhine primates, but not for catarrhine primates, as these two taxa have also been found to differ with regard to other aspects of sweet-taste perception, e.g., the ability to detect certain types of proteins that taste sweet to humans (Glaser et al. 1978, 1992, 1996).

**Table 3:** Taste preference difference thresholds, expressed as Weber ratios (Δ*I*/*I*), for sweet-tasting substances in primates and in non-primate mammals

Species	Weber ratio	Substance	References
Hominid primates			
*Pan troglodytes verus*	0.30–0.40	Sucrose	[1]
*Homo sapiens*	0.06–0.12	Sucrose	[2]
*Homo sapiens*	0.13–0.16	Sucrose	[3]
*Homo sapiens*	0.15–0.18	Sucrose	[4]
*Homo sapiens*	0.14	Sucrose	[5]
*Homo sapiens*	0.17	Sucrose	[6]
*Homo sapiens*	0.14–0.30	Sucrose	[7]
*Homo sapiens*	0.13–0.50	Sucrose	[8]
*Homo sapiens*	0.34–0.44	Sucrose	[9]
*Homo sapiens*	0.06–0.28	Glucose	[10]
*Homo sapiens*	0.16–0.27	Saccharin	[8]
*Homo sapiens*	0.26–0.38	Saccharin	[9]
Catarrhine primates			
*Papio hamadryas anubis*	0.10–0.25	Sucrose	[11]
Platyrrhine primates			
*Saimiri sciureus*	0.30–0.50	Sucrose	[12]
*Saimiri sciureus*	0.33	Glucose	[13]
*Ateles geoffroyi*	0.075–0.25	Sucrose	[11]
Non-primate mammals			
*Rattus norvegicus*	0.11–0.15	Saccharin	[14]
*Rattus norvegicus*	0.37	Glucose	[15]

[1] present study; [2] Lundgren et al. (1976); [3] Gilmore and Murphy (1989); [4] Laing et al. (1993); [5] McBride (1983); [6] Geldard (1972); [7] Schutz and Pilgrim (1957); [8] Pfaffmann et al. (1971); [9] Fischer et al. (1965); [10] Berg et al. (1955); [11] Laska et al. (1999a, b); [12] Laska (1994); [13] Wagner et al. (1965); [14] Brosvic and Slotnick (1986); [15] Scott and Giza (1987)

The taste difference threshold values for sucrose reported in human studies show considerable variation and a general tendency for lower values compared to the chimpanzees studied here (see Table 3). Whereas the former is probably due to the fact that the human studies employed different methods (e.g., triangle or paired-comparison tests) and threshold criteria, the latter can probably be ascribed to the fact that signal detection methods yield lower threshold values compared to simple two-bottle preference tests (Spector 2003). In this context, it should be mentioned that all animal data reported in Table 3 are based on the same method, that is, two-bottle preference tests of short duration. The findings of the present study do not support the notion that dietary specialization would systematically affect taste preference difference thresholds.

Taken together, the results of the present study are in line with the notion that taste sensitivity for food-associated carbohydrates may correlate positively with phylogenetic relatedness. Further, they support the notion that relative preferences for food-associated carbohydrates, but not taste preference difference thresholds, may correlate with dietary specialization in primates.
